# A genetic cluster of MDR *Enterobacter cloacae* complex ST78 harbouring a plasmid containing *bla*_VIM-1_ and *mcr-9* in the Netherlands

**DOI:** 10.1093/jacamr/dlab046

**Published:** 2021-05-12

**Authors:** Antoni P A Hendrickx, Sylvia Debast, María Pérez-Vázquez, Annelot F Schoffelen, Daan W Notermans, Fabian Landman, Cornelia C H Wielders, Javier E Cañada Garcia, Jacky Flipse, Angela de Haan, Sandra Witteveen, Marga van Santen-Verheuvel, Sabine C de Greeff, Ed Kuijper, Leo M Schouls, Dutch CPE surveillance Study Group, A Maijer-Reuwer, M A Leversteijn-Van Hall, J A J W Kluytmans, I J B Spijkerman, K Van Dijk, T Halaby, B Zwart, B M W Diederen, A Voss, J W Dorigo-Zetsma, A Ott, J H Oudbier, M Van der Vusse, A L M Vlek, A G M Buiting, L Bode, S Paltansing, A J Van Griethuysen, M Den Reijer, M Van Trijp, E P M Van Elzakker, A E Muller, M P M Van der Linden, M Van Rijn, M J H M Wolfhagen, K Waar, E Kolwijck, W Silvis, T Schulin, M Damen, S Dinant, S P Van Mens, D C Melles, J W T Cohen Stuart, M L Van Ogtrop, I T M A Overdevest, A P Van Dam, H Wertheim, H M E Frénay, J C Sinnige, E E Mattsson, R W Bosboom, A Stam, E De Jong, N Roescher, E Heikens, R Steingrover, A Troelstra, E Bathoorn, T A M Trienekens, D W Van Dam, E I G B De Brauwer, F S Stals

**Affiliations:** 1 Center for Infectious Disease Control (CIb), National Institute for Public Health and the Environment (RIVM), Bilthoven, The Netherlands; 2 Isala, Laboratory for Medical Microbiology and Infectious Diseases, Zwolle, The Netherlands; 3 Laboratorio de Referencia e Investigación en Resistencia a Antibióticos e Infecciones Relacionadas con la Asistencia Sanitaria, Centro Nacional de Microbiología, Instituto de Salud Carlos III, Majadahonda, Madrid, Spain

## Abstract

**Background:**

Carbapenemases produced by Enterobacterales are often encoded by genes on transferable plasmids and represent a major healthcare problem, especially if the plasmids contain additional antibiotic resistance genes. As part of Dutch national surveillance, 50 medical microbiological laboratories submit their Enterobacterales isolates suspected of carbapenemase production to the National Institute for Public Health and the Environment for characterization. All isolates for which carbapenemase production is confirmed are subjected to next-generation sequencing.

**Objectives:**

To study the molecular characteristics of a genetic cluster of *Enterobacter cloacae* complex isolates collected in Dutch national surveillance in the period 2015–20 in the Netherlands.

**Methods:**

Short- and long-read genome sequencing was used in combination with MLST and pan-genome MLST (pgMLST) analyses. Automated antimicrobial susceptibility testing (AST), the Etest for meropenem and the broth microdilution test for colistin were performed. The carbapenem inactivation method was used to assess carbapenemase production.

**Results:**

pgMLST revealed that nine *E. cloacae* complex isolates from three different hospitals in the Netherlands differed by <20 alleles and grouped in a genetic cluster termed EclCluster-013. Seven isolates were submitted by one hospital in 2016–20. EclCluster-013 isolates produced carbapenemase and were from ST78, a globally disseminated lineage. EclCluster-013 isolates harboured a 316 078 bp IncH12 plasmid carrying the *bla*_VIM-1_ carbapenemase and the novel *mcr-9* colistin resistance gene along with genes encoding resistance to different antibiotic classes. AST showed that EclCluster-013 isolates were MDR, but susceptible to meropenem (<2 mg/L) and colistin (<2 mg/L).

**Conclusions:**

The EclCluster-013 reported here represents an MDR *E. cloacae* complex ST78 strain containing an IncH12 plasmid carrying both the *bla*_VIM-1_ carbapenemase and the *mcr-9* colistin resistance gene.

## Introduction

Carbapenems are a class of antibiotics often used as a last resort. In carbapenemase-producing Enterobacterales (CPE), genes conferring resistance to carbapenem antibiotics have spread globally, typically through transmissible plasmids.^1^ From 2014 until 2019, the predominant CPE species in the Netherlands were *Klebsiella pneumoniae* (43%), *Escherichia coli* (30%) and *Enterobacter cloacae* complex (13%).^2^ Common carbapenemase resistance genes are the Verona integron-encoded metallo-β-lactamase (VIM)-type of carbapenem hydrolysing enzymes that belong to the Ambler class B type of carbapenemases.^3^ Plasmids that harbour a *bla*_VIM-1_ integron belong to the IncA/C family, which can be transferred between bacteria via conjugation and have been described in various CPEs.^4^ Colistin is considered as a rescue agent for the treatment of MDR CPE and acquired resistance may have important consequences. Recently, a novel plasmid-mediated colistin resistance gene, termed *mcr-9*, was described.^5^ Mobile colistin resistance ‘*mcr*’ genes encode phosphoethanolamine transferases that modify the lipid A moiety of lipopolysaccharide, ultimately leading to colistin resistance.^6^ Thus far, 10 different *mcr* genes have been described in a variety of Gram-negative organisms including Enterobacterales, *Acinetobacter* spp. and *Pseudomonas* spp.^5^^,^^7–15^ Plasmids containing *mcr* genes carry a variety of replicons, and until now *mcr-9* has only been associated with plasmids harbouring the IncH12 replicon. Here we report on nine genetically highly related *E. cloacae* complex isolates harbouring both the *bla*_VIM-1_ and *mcr-9* genes, which were found in three different Dutch hospitals in the Dutch national CPE surveillance.

## Methods

### Antimicrobial susceptibility testing of bacterial isolates

In the national CPE surveillance, 50 medical microbiology laboratories from the Netherlands send CPE isolates with a meropenem MIC of ≥0.25 mg/L and/or an imipenem MIC of ≥1 mg/L or phenotypic/genotypical evidence of carbapenemase production to the National Institute for Public Health and the Environment (RIVM).^16^ In this study, 224 unique *E. cloacae* complex isolates were included from the national CPE surveillance in the period 2011–20. For all 224 isolates the MIC for meropenem was determined by Etest, and carbapenemase production was assessed by the carbapenem inactivation method (CIM) (Supplementary data, available at *JAC-AMR* Online).^17^ Broth microdilution (BMD) was used to determine colistin resistance. Hospital-1 performed automated AST on EclCluster-013 isolates.

### Next-generation and third-generation sequencing

Illumina next-generation sequencing (NGS) of all 224 *E. cloacae* complex isolates, Nanopore third-generation sequencing (TGS) of the nine EclCluster-013 isolates, and plasmid reconstructions were performed as described previously.^16^ Resistome and replicome analyses of the EclCluster-013 isolates were performed using ResFinder (v3.1.0) and PlasmidFinder (v 2.0.2) software as described previously.^16^ NGS sequence data are available at ENA (PRJEB41940), and plasmid and chromosome sequences in GenBank (PRJNA695145).

### MLST and pan-genome MLST (pgMLST)

The NGS data of the *E. cloacae* isolates were used for classical MLST and pgMLST analyses (SeqSphere v6.0.2, BioNumerics v7.6.3). The minimum spanning tree (MST) was based on an in-house *E. cloacae* complex pgMLST scheme comprised of 9829 genes from the reference CP001918, CP017186 and CP017184 isolates. pgMLST clusters were defined as ≥2 isolates of which the genetic distance was ≤20 alleles (20/9829 ≤ 0.2% different).

## Results

### Genetic cluster of E. cloacae complex carrying bla_VIM-1_ and mcr-9

**Figure 1.**
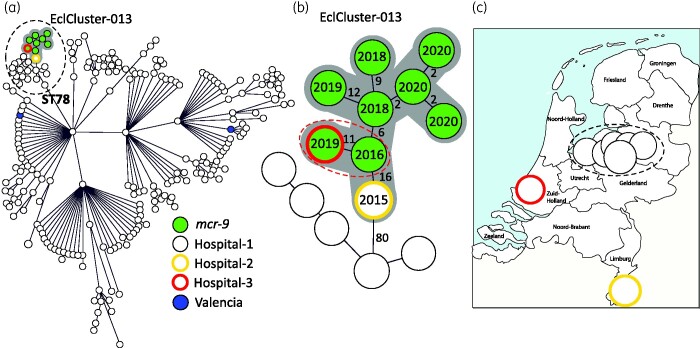
pgMLST analysis of *E. cloacae* EclCluster-013. (a) Minimum spanning tree containing nine *bla*_VIM-1_ positive *E. cloacae* isolates which form a genetic cluster (encircled with dotted line and with a grey halo) in the *E. cloacae* complex population. A genetic cluster is defined as ≥2 isolates that differ by ≤20 alleles. The *mcr-9* colistin resistance gene is in green. Black, yellow and red circles indicate the three different hospitals. Blue indicates isolates obtained from the autonomous community of Valencia, Spain. (b) In-depth analysis of the allelic difference between the nine isolates. The allelic difference is indicated as numbers on the lines and year of isolation is indicated on the circles. The red dotted line indicates patients with travel history to Spain. (c) Geographic distribution of *E. cloacae* complex *bla*_VIM-1_ in the Netherlands.

**Figure 2.**
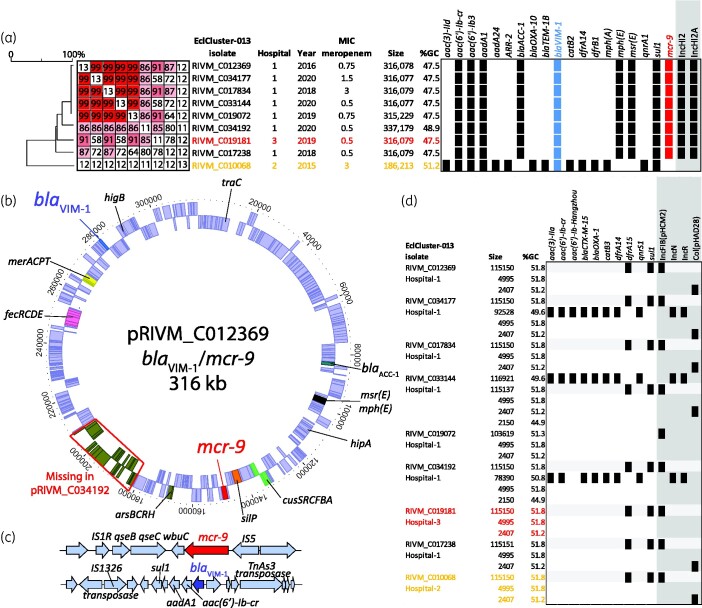
The resistome of EclCluster-013 and the *bla*_VIM-1_ and *mcr-9* plasmid. (a) UPGMA hierarchical clustering combined with the percentage plasmid DNA similarity among the plasmids. The presence of AMR genes is indicated with squares. Blue squares indicate *bla*_VIM-1_, red squares the *mcr-9* resistance genes and black squares additional AMR genes, and the replicons are indicated in grey. Plasmid size is in bp. MIC is in mg/L. (b) Structure of the *bla*_VIM-1_ and *mcr-9* plasmid of *E. cloacae* complex with functional groups indicated. (c) Architecture of the putative *mcr-9* mobile genetic element and *bla*_VIM-1_ integron of EclCluster-013. (d) Resistome of additional plasmids of EclCluster-013 isolates.

Since 2014, the Dutch national CPE surveillance programme of the RIVM has collected CPE that are found in 50 participating medical microbiology laboratories. Received isolates are routinely sequenced using NGS and TGS. In the Netherlands, *E. cloacae* complex ranked third among most commonly found CPE species in 2014–19.^2^ Nine *E. cloacae* isolates submitted in the period from January 2015 until September 2020 belonged to a genetic cluster designated as EclCluster-013 (Figure 1a). This EclCluster-013 is comprised of VIM-1-producing multiresistant *E. cloacae* complex from MLST ST78, a high-risk and globally spread genetic lineage.^18^ Based on pgMLST analysis using an in-house pgMLST scheme, the nine isolates differed by 20 alleles (20/9829; 0.2%) or less, demonstrating that the nine isolates belonged to a genetic cluster (Figure 1a). More specifically, in-depth analysis to determine the genetic relatedness among the nine isolates revealed that the Hospital-1 isolate from 2016 differed in 16 (0.16%) and 11 (0.11%) alleles from the Hospital-2 and Hospital-3 isolates, respectively. The most recent isolates obtained in 2020 from Hospital-1 differed by only 2 alleles (0.02%) from each other (Figure 1b). The nine isolates were obtained from nine patients and were sent to the RIVM by three hospitals (Figure 1c). Of the nine isolates, seven were submitted by one hospital in the period 2016–20. In six patients, the cultures were taken for the purpose of screening for MDR organisms upon admission and those patients were colonized with CPE without having an infection. Three isolates were retrieved from patients with a presumed respiratory tract infection and catheter-associated urinary tract infection, respectively. All EclCluster-013 isolates were positive for the production of carbapenemase according to the CIM,^17^ and the MIC for meropenem was low (Figure 2a). Epidemiological information revealed that two of the nine patients from different hospitals had been admitted to a hospital in Benidorm in Spain within the 2 months prior to patient sampling in 2016 and 2019, which is considered as a risk factor for the acquisition of CPE. For the other seven patients in the cluster, no known risk factors related to CPE carriage were reported. In eight of the nine isolates the *mcr-9* colistin resistance gene was found in addition to *bla*_VIM-1_, among other antibiotic resistance genes (Figures 1a and 2a). To further investigate the connection with Spain, two *bla*_VIM-1_- and *mcr-9-*containing *E. cloacae* complex isolates were retrieved from the autonomous community of Valencia in Spain. pgMLST analysis demonstrated that these two isolates were unrelated to EclCluster-013 since these isolates belonged to MLST ST182 and ST566 (Figure 1a).

### A bla_VIM-1_ and mcr-9 carrying plasmid in E. cloacae complex

Both the *mcr-9* and *bla*_VIM-1_ genes were present on a new 316 kb plasmid from the IncH12 replicon family, which was not reported in the National Center for Biotechnology Information database (NCBI). Comparison of the 316 kb plasmid with recently reported *bla*_VIM_/*mcr-9* plasmids from NCBI showed only low similarity (Supplementary data). The plasmid was 87%–99% identical and had a percentage G + C content of 47.5%, except in the isolate from Hospital-2 lacking *mcr-9* (Figure 2a). The plasmid of the Hospital-2 isolate displayed only 12% similarity, had an aberrant size, percentage G + C content and resistome, had a distinct *bla*_VIM-1_ integron and lacked the IncH12 replicon, and thus represents a different plasmid. The plasmid of the Hospital-3 isolate RIVM_C019181 is 316 kb and highly resembled the 316 kb plasmid of RIVM_C012369. The plasmid of 337.2 kb from the RIVM_C034192 isolate lacked a ∼27 kb fragment but acquired a ∼48 kb element in the same position. The 316 kb plasmid also contained genes encoding resistance to different classes of antibiotic, including aminoglycosides [*aac(6’)Ib, aac(6’)-Ib-cr*], β-lactams (*bla*_VIM-1_, *bla*_ACC-1_), fluoroquinolones [*aac(6’)-Ib-cr*], macrolides [*mph(E)*], sulphonamide (*sul1*), and streptogramin B [*msr(E)*] (Figure 2a). The fosfomycin (*fosA*) gene was located on the chromosome in all isolates. Automated AST confirmed this MDR phenotype (Supplementary data). The *mcr-9* colistin resistance gene was localized within a putative mobile genetic element in the plasmid and comprised *qseB-qseC-wbuC-mcr-9* flanked by IS*1R* and IS*5* insertion elements (Figure 2b and c). The *mcr-9* gene was one codon longer than the ResFinder reference *mcr-9* gene, supplying the phosphoethanolamine transferase protein with an additional C-terminal tryptophan. The *qseB-qseC* genes are suggested to represent a two-component system implicated in the induction of colistin resistance, possibly leading to resistance to colistin during colistin treatment of patients with an *E. cloacae* infection. The BMD test according to EUCAST guidelines for colistin resistance of the EclCluster-013 isolates indicated that all isolates were susceptible to colistin (MIC <2 mg/L) and remained so even after repeated re-culturing of sub-MIC grown cells to induce colistin resistance. The sulphonamide resistance gene *sul1* is localized together with *dfrA15* on a different resistance plasmid of 115 kb with the IncFIB replicon (Figure 2d). Three of the nine EclCluster-013 isolates had additional medium-sized plasmids from different IncR/IncN replicon families, which impacted the resistome. The 316 kb plasmid also contained conjugation machinery, two toxins (HipA and HigB), *virB* type IV secretion system and multiple gene clusters implicated in iron (*fieF*), cation (*cusSRCFBA*), Fe^3+^ dicitrate (*fecRCDE*), arsenic (*arsBCRH*), mercury (*merACPT*), chromate (*srpC*) and silver (*silPE*) heavy metal transport (Figure 2b).

## Discussion

Here we report a genetic cluster of an MDR *E. cloacae* complex ST78 containing the *bla*_VIM-1_ carbapenemase and the *mcr-9* colistin resistance gene on an antibiotic resistance plasmid with the IncH12 replicon. Although two of the patients had been admitted to a Spanish hospital, the *bla*_VIM-1_/*mcr-9*-carrying isolates from Spain and the Netherlands differed considerably, failing to confirm transmission. The origin and reservoir of both the 316 kb plasmid and the ST78 strain remain to be elucidated and represent a limitation of this study. Possibly, there is a hidden endemicity of this *E. cloacae* complex ST78 cluster independent of the acquisition of the *bla*_VIM-1_ plasmid. Colistin is not commonly used in the clinical practice in the Netherlands, but it is administered to patients with MDR CPE infections or during selective digestive decontamination in ICUs in hospitals, representing a potential risk of acquisition and selection of colistin-resistant *E. cloacae*.^19^ While there was no phenotypic colistin resistance detected by BMD in the multiresistant isolates carrying the *mcr-9* gene as also observed by others,^20^ the resistance plasmid may be transferable to other CPEs, such as *K. pneumoniae* or *E. coli* where *mcr-9* can cause colistin resistance.^5^ The data presented here suggest that the presence of the *mcr-9* gene in *E. cloacae* complex does not lead to colistin resistance, nor is it induced under laboratory conditions.

## Supplementary Material

dlab046_Supplementary_Data
